# “Best fit” framework synthesis: refining the method

**DOI:** 10.1186/1471-2288-13-37

**Published:** 2013-03-13

**Authors:** Christopher Carroll, Andrew Booth, Joanna Leaviss, Jo Rick

**Affiliations:** 1 Health Economics and Decision Science (HEDS), School of Health and Related Research (ScHARR), Regent Court, Regent Street, Sheffield, S1 4DA, UK; 2 Health Sciences Research Group - Primary Care, School of Community Based Medicine, University of Manchester, Williamson Building, Manchester, M13 9PL, UK

**Keywords:** Systematic review, Qualitative research, Methods, Framework synthesis, Thematic analysis, Sensitivity analysis, Smoking cessation, Critical appraisal, Theory

## Abstract

**Background:**

Following publication of the first worked example of the “best fit” method of evidence synthesis for the systematic review of qualitative evidence in this journal, the originators of the method identified a need to specify more fully some aspects of this particular derivative of framework synthesis.

**Methods and Results:**

We therefore present a second such worked example in which all techniques are defined and explained, and their appropriateness is assessed. Specified features of the method include the development of new techniques to identify theories in a systematic manner; the creation of an *a priori* framework for the synthesis; and the “testing” of the synthesis. An innovative combination of existing methods of quality assessment, analysis and synthesis is used to complete the process. This second worked example was a qualitative evidence synthesis of employees’ views of workplace smoking cessation interventions, in which the “best fit” method was found to be practical and fit for purpose.

**Conclusions:**

The method is suited to producing context-specific conceptual models for describing or explaining the decision-making and health behaviours of patients and other groups. It offers a pragmatic means of conducting rapid qualitative evidence synthesis and generating programme theories relating to intervention effectiveness, which might be of relevance both to researchers and policy-makers.

## Background

The technique of "best fit" framework synthesis was described in a previous paper in this journal [1]. The "best fit" framework synthesis method offered a means to test, reinforce and build on an existing published model, conceived for a potentially different but relevant population. As with similar approaches, it involved an examination of existing relevant theories, “their testability, falsifiability, their internal logic and their fit with the evidence” [2].

“Best fit” framework synthesis begins by creating a framework of *a priori* themes and coding data from a review’s included studies against that thematic or conceptual framework. This approach produces a relatively rapid, transparent and pragmatic process [3] when compared to more exclusively interpretative forms of synthesis because a substantial amount of the data to be included in the review is often coded against the *a priori* framework. Only data that cannot be accommodated within the framework requires considered, iterative interpretation using inductive, thematic analysis techniques. The approaches to synthesis are therefore both positivist and interpretive [4]; it harnesses the recognised strengths of both framework and thematic synthesis [5].

This methodology is different from other approaches to qualitative evidence synthesis in part because it employs a systematic method for identifying published frameworks, models or theories in order to create the framework for the synthesis. It is also different because it combines both framework and thematic analysis techniques to complete the synthesis. The potential value of the “best fit” method was quickly recognised, especially for qualitative evidence synthesis to address “policy-urgent” questions [3,6,7]. This was because "both thematic synthesis and framework synthesis – while … involving some interpretation of data – share a … less problematized view of reality and a greater assumption that their synthetic products are reproducible and correspond to a shared reality … directly applicable to policy makers and designers of interventions", while approaches such as meta-ethnography and critical interpretive synthesis are "generally more complex and conceptual, sometimes operating on the symbolic or metaphorical level, and requiring a further process of interpretation by policy makers and practitioners in order for them to inform practice” [5].

The previous paper described the first attempt at this form of framework synthesis. Subsequently its originators identified a need to specify and extend, methodologically, some aspects of the process. These include the identification of the foundation theory, the thematic reduction of this theory to create the *a priori* framework, and the transition from the resultant framework (based on the *a priori* framework plus new themes) to the final conceptual model. This paper therefore defines the processes for each of these stages, as well as providing a further opportunity to apply and evaluate the original data extraction, quality assessment and synthesis processes.

The aim is to provide complete transparency for all of these constituent stages and processes, a key characteristic of systematic review and evidence synthesis, but one frequently lacking for some qualitative synthesis methods. These methods often might fail to specify how to identify and select a relevant theory [8] or what method to apply to analyse data or evidence that do not fit into an *a priori* framework [9-13]. Such omissions have been noted previously, especially in relation to the conduct of meta-ethnography [9], which is the most frequently-conducted type of qualitative evidence synthesis [14].

The sample case study here is a qualitative evidence synthesis of the views and preferences of employees regarding workplace strategies or interventions to reduce smoking or facilitate smoking cessation [15]. Opportunism dictated this choice of case study, i.e. a piece of work was commissioned from two of the authors (CC, JR) focusing on an aspect of health behaviour change (smoking cessation) and this offered the opportunity to conduct a qualitative evidence synthesis.

### Methods

**Figure 1:**
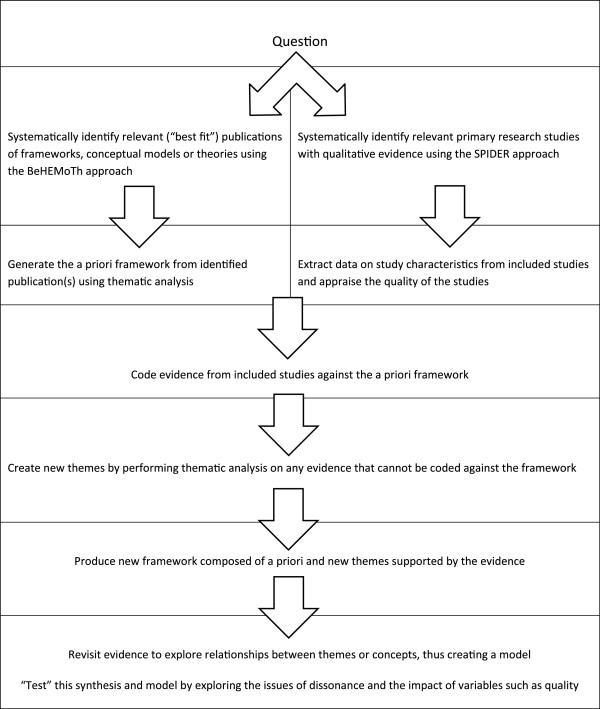
Qualitative evidence synthesis using “Best-fit” framework synthesis.

“Best fit” framework synthesis requires identification of a relevant framework, theory or conceptual model for particular health behaviours. This is then reduced to its key elements or variables, which form the themes of the *a priori* framework. Primary research studies for inclusion in the review are identified and selected by applying conventional systematic review methods. Evidence from these included studies is then coded against the themes of the *a priori* framework and new themes are generated from evidence not captured by this *a priori* framework. These new themes are based on the reviewers’ interpretation of the evidence and constant comparison of such new themes across studies. The principles of the method correspond to those for the thematic analysis of primary research data, e.g. transcripts of interviews, as described by Miles and Huberman [16], but are applied to the findings or results reported in published papers; hence, secondary thematic analysis. Relationships between the themes of the framework are then either recreated or generated based on the evidence from the primary research studies included in the review. A new model or theory of the particular health behaviour of interest in the population or setting of interest is thus created. The process is outlined in Figure 1. Once the question is determined, the creation of the *a priori* framework for the synthesis is conducted simultaneously with but independently from the search for and selection of the primary research studies to be included in the review and synthesis. These two “strands” then join together at the framework synthesis stage. In the case study considered here, the resultant framework and model represented employees’ views about, and their experiences of work-based smoking cessation programmes.

The method requires two separate sets of inclusion criteria, searches and study selection: One for identifying the models and theories to generate the *a priori* framework, and one for populating the systematic review of primary qualitative research studies (Table 1).

**Table 1:** Inclusion criteria

	**Models and theories**	**Primary research studies**
**Setting/Population**	Workplace or employees	Workplace
**Phenomenon of interest**	Smoking reduction, cessation or restriction	Smoking reduction, cessation or restriction
**Design, Evaluation, Research**	Publications exploring, testing or creating frameworks, models, theories	Interviews, focus groups, or satisfaction surveys (that quantify employees’ views, attitudes or preferences in terms of frequencies)

### Inclusion criteria

#### Searching and study selection

##### Identification of relevant models and theories

Other forms of framework synthesis have developed an *a priori* framework for the analysis through a combination of consultation, literature review, and consensus [10,13] or based it upon the most common model in the included studies [12,17]. In the original worked example of “best fit” one of the authors (AB) identified the foundation theory or model from a grey literature conceptual model following iterative searching of bibliographic databases and Internet search engines. The authors acknowledged that lack of transparency was a weakness of this approach. If the approach was to be reproducible and usable for others then a clearly-defined, systematic means for identifying relevant published models or theories was required, from which to generate the *a priori* framework for the synthesis. The authors have subsequently developed such a strategy and submitted it for publication elsewhere (Booth A, Carroll C: Towards a simple transparent method for identifying theory for use in systematic reviews of behaviour change interventions: the BeHEMoTh Procedure. Submitted.). This BeHEMoTh strategy (named as a mnemonic from the component elements Behaviour of Interest, Health context, Exclusions and Models or Theories) provides a multi-stage, systematic approach to identifying relevant models and theories. In this case study it was only necessary to use the first stage of the process, that is, combining free text and database thesaurus terms for the behaviour of interest (smoking cessation) and health context (workplace), with terms for models and theories (see Table 2). This was because this approach generated five equally relevant conceptual papers for use in creating the *a priori* framework (see below); progress to the additional stages would have been undertaken if none or perhaps only one such publication was identified, potentially compromising the creation of the framework.

**Table 2:** Search strategy following BeHEMoTh approach

**Strategy**	**Terms**
**Be** - Behaviour of Interest:	Smoking cessation or health promotion
**H** - Health Context	Workplace
**E** - Exclusions	Regression or integrative model or integrative care model or economic or Markov or animal
**MoTh** - Models or Theories	Model or theory or theories or framework or concept or conceptual

Search strategy: (Be AND H AND MoTh) NOT E.

The search for models to inform the exemplar review included terms for workplace health promotion (WHP) as well as workplace smoking cessation, in order to be as sensitive as possible and to identify WHP research that included smoking cessation, but might not actually specify it in title or abstract (see Additional file 1). This broad approach was adopted to anticipate a circumstance where no model specifically relating to workplace smoking cessation can be identified from the literature. In accordance with emerging practice in the field of health services research, where core databases judiciously selected for the topic and study types of interest might be considered sufficient to retrieve a critical majority of the relevant literature when operating under time or resource constraints [18,19], the following three databases were interrogated by the lead author: PsycINFO, CINAHL and MEDLINE. More recent research suggests that the Social Sciences Citation Index represents either an additional potential source in this search or a potential substitute for CINAHL [20].

**Figure 2:**
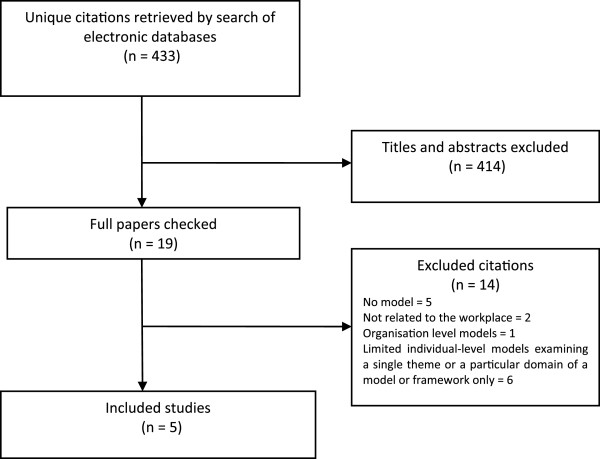
PRISMA flowchart of frameworks, models and theories search.

The first author screened all titles and abstracts of citations retrieved by the search to identify models or theories appropriate to this review, as defined by the inclusion criteria. The search generated 433 unique citations from across the three databases. Full papers of potentially relevant citations were retrieved and checked for relevance. From these citations, the lead author identified five publications with relevant models or theories that seemed to represent a good “fit” to the population, setting and health behaviour of interest (i.e. the inclusion criteria): Employee attitudes and responses regarding smoking cessation or reduction in the workplace. For the PRISMA flowchart, see Figure 2. This provided corroboration that the BeHEMoTh strategy is a feasible means of systematically identifying relevant models and theories. Reference lists of all papers satisfying the inclusion criteria were also checked for additional relevant citations.

##### Primary research studies for the qualitative synthesis

An evaluated, published search strategy for identifying primary qualitative research studies was used to identify studies for inclusion in the qualitative review: SPIDER (Sample, Phenomenon of Interest, Design, Evaluation and Research type) [21]. This strategy involved combining free text and database thesaurus terms for workplace or employees with terms for smoking cessation or health promotion, and terms for qualitative research. As above, the search also included terms for workplace health promotion (WHP) (see Additional file 1). The following databases were interrogated to identify relevant occupational health and social science literature, both published and unpublished: Social Science Citation Index, PsycINFO, CINAHL, ASSIA, IBSS, Emerald reviews, ERIC and MEDLINE. All searches were conducted by the first author, a qualified information specialist. The choice of sources was determined by the potential “scatter” of the topic’s research literature across multiple fields and databases [20,22,23], i.e. the literature was likely to be found in resources for medicine, health, psychology, social science and even education, rather than a single field, such as medicine alone. Independent screening of all citations was conducted by two reviewers (JL, JR). Reference lists of all papers satisfying the inclusion criteria were also checked for additional relevant citations.

**Figure 3:**
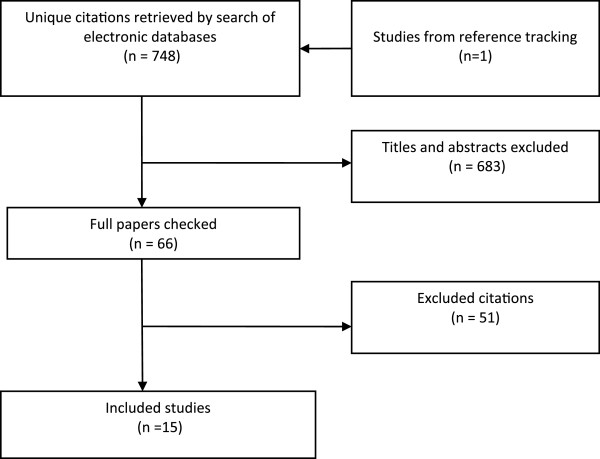
PRISMA flowchart of primary research studies search.

The search generated 748 unique citations from across eight databases. Sixty-five full papers were retrieved as potentially relevant, of which 14 studies were found to satisfy the inclusion criteria. One additional relevant study was identified from reference lists [24]. For the PRISMA flowchart, see Figure 3. Six of the included studies examined people’s views about employer’s decisions to restrict smoking within or at the workplace [24-29]; five explored views relating to complex interventions, i.e. involving a combination of at least two or more of the following: self-help or educational materials, smoking cessation resources or “props” such as nicotine patches or pencil cigarettes, support groups, peer support, telephone counselling, and competitions or incentives [30-34]; one employed telephone counselling only [35] and one incentives only [36]. Two studies did not specify an intervention [37,38], but rather elicited people’s views on the principle of a workplace smoking cessation intervention.

#### Data extraction

##### Models and theories

Five papers satisfied the inclusion criteria (Table 1 above) for informing the *a priori* framework. Each of the five papers presented a relevant conceptual model adapted from, or using in part, modified versions of one of three principal foundation models: The Transtheoretical Model (TTM) of Behaviour Change, including its related Stages and Processes of Change elements [39], the Theory of Planned Behaviour (TPB) [40] and the Health Belief Model (HBM) [41]. Three papers reported conceptual models based on the TTM [42-44], one on the TPB [45] and one on the HBM [46]. In the previous worked example, only a single conceptual model was identified for developing the *a priori* framework. In this new case study, however, the review team were faced with three equally relevant models with variants. The options were to privilege one of the models (i.e. to choose one particular model and reject the others, arbitrarily or using *post hoc* criteria), use two or more models in combination, or to produce an *a priori* framework based on all five publications. The third option was selected. This was done because there was no empirical justification for the selection of one model over another in this review: All of the conceptual models were considered to be relevant, i.e. satisfying the inclusion criteria, and, in combination, potentially offered a more comprehensive foundation for the synthesis than might be possible with a single, arbitrarily-chosen model.

**Figure 4:**
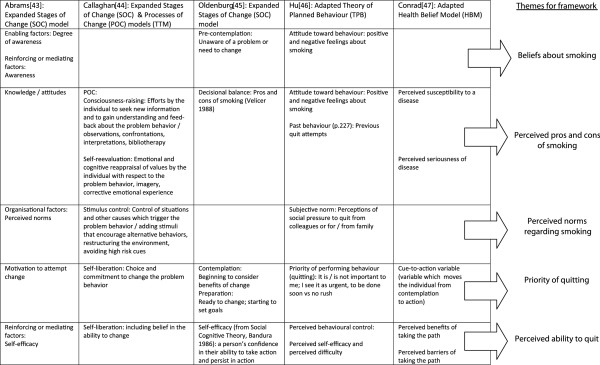
From the models to the framework of themes.

Thematic analysis was chosen for creating the *a priori* framework from the five publications as it is a widely-used form of inductive analysis and is consistent with the final synthesis process for the “best fit” method. This method of analysis works by identifying commonalities and differences between the models or theories and naming them as themes [16]. These themes form the *a priori* framework for the synthesis. This process is represented in Figure 4. Each theme was then supported with a definition based on the elements in the original papers, thus creating “concepts” [47], in order to facilitate the coding of data against this framework. This step was taken because experience from the original worked example suggested that clearly-defined concepts are required to enable independent reviewers to code consistently, without interpreting the same named theme differently [1]. The resultant framework for coding data extracted from included primary studies, based on the results of this thematic analysis of models is given in Table 3. The data extracted from studies identified for the review were coded against these concepts in the data extraction form.

**Table 3:** The coding framework

**Concepts derived for coding**	**Definitions**
Beliefs about smoking	Person considers there to be or not to be a problem
Perceived pros and cons of smoking	Person beginning to consider benefits of change;
	Perceived susceptibility to disease (I don’t think anything will happen to me *vs* my family has a history)
	Perceived seriousness of disease (not bothered *vs* very concerned)
Perceived norms regarding smoking	I am participating or not participating because it is expected of me
Priority of quitting	It is/is not important to me; I see it as urgent, to be done soon *vs* no rush
Perceived ability to quit	A person’s confidence in their ability to take action and persist in action: I feel able to quit or I feel the programme provides me the ability or motivation to quit; self-efficacy
Dependence	I am addicted, nothing will work; or no programme works; I’ve tried quitting before but without success, it’s too hard
Social support	It was very helpful to have the support of my: Friends; Family
Organisation support	The work environment is/is not conducive to quitting smoking
Opportunity	I am participating because the programme is available
Substitutes	Substitution of alternatives to the problem behaviour
Incentives to quit	Receiving a reward for making the change. The provision of items such as money, prizes and products, or some form of self-reward, which are intended to motivate smokers to reduce consumption or quit

##### Primary research studies

The data extraction form for the primary research studies derived its elements from three sources. First, it was based on the key data required for the synthesis and its interpretation, such as details of the population, setting and intervention. Second, it also included the framework concepts generated from thematic analysis of the models, as described above. The data for analysis were extracted from the Results sections of papers and consisted either of verbatim quotations from study participants or findings reported by authors that were clearly supported by study data. Three reviewers independently piloted the form on two studies, before a final, agreed form was achieved. After a check had been made for consistency of extraction across two included studies, two reviewers (JL, JR) each independently coded the results data for all papers against the *a priori* concepts derived from the relevant conceptual model. They also independently generated new themes for evidence or findings that could not be accommodated by the *a priori* framework, as described below.

Finally, the data extraction form also included quality assessment criteria. The two reviewers conducted independent quality assessments of the included studies using published criteria [48], which focused on how the design and conduct of each study had been reported, rather than necessitating potentially more subjective judgements on less easily apprehended characteristics. These assessments were used to inform judgments on both the internal validity of the studies and, consequently, the validity of the findings of the synthesis.

#### Synthesis and the conceptual model

The final list of concepts was synthesised, with reference to the extracted data from the included studies, to construct a new, evidence-based conceptual model regarding employees’ views of workplace smoking cessation programmes and policies. This was completed in two stages. First, a new conceptual framework is created composed of a simple list of defined themes consisting of any *a priori* themes supported by evidence from the included studies, plus any new themes generated by thematic analysis of evidence falling outside of the framework (such as Tables 3 and 4). The relationships between these themes are neither apparent nor detailed in such a framework, rather the themes exist as discrete elements within it. The evidence underpinning the framework is then revisited and relationships between the framework’s themes are established and illustrated. These stages are detailed below.

**Table 4:** New concepts from the secondary thematic analysis

**Concepts derived for coding**	**Definitions**
**Employees’ expectations of employees**	
Obligations	The necessity for employers to comply with formal regulations regarding the law on smoking bans or restrictions
Responsibilities	The non-legal responsibilities of employers regarding smoking restrictions or cessation. These might concern either protection for non-smokers or help for smokers
Enforcement	Employees’ experience regarding whether or not legal or other regulations are actually enforced
**Intervention preferences**	
Ease and convenience	The accessibility both of the self-help materials and other types of support, such as counselling or groups
Alternatives* and cost	The provision of, and problems associated with such alternatives, such as cost
Co-worker support	The use of co-workers within the intervention, just as peer support, support groups, and the institutional encouragement of interventions creating a shared experience

*Alternatives also existed as an *a priori* theme but was refined and extended as a result of the analysis.

##### Stage 1: The creation of a new conceptual framework

The process of data extraction and coding led to the slight revision of three existing concepts in the framework: *Organisation support* was re-specified as *Employer support*, and *Social Support* as *Co*-*worker interaction* (*workplace*) and *Social Context* (*non*-*workplace*), and *Alternatives* as *Alternatives and Cost*, in order to capture more accurately the relationship or concept as described by participants in the studies. Data from the included studies were found to support all concepts in the *a priori* framework, i.e. none was dropped from the final synthesis because of an absence of evidence to support it. It is worth pointing out that this was the case even though none of the interview schedules or coding in the included primary research studies was structured explicitly around any of the models identified for the framework. All of the key concepts derived from the foundation models therefore resonated with the data of studies included in this review. These included employees’ awareness or beliefs regarding problems with smoking, pros and cons and perceived norms regarding it, and the factors mediating the relationship between any intervention and successful quitting: Dependence, priority of quitting and self-efficacy (perceived ability to quit), and some aspects of interventions, such as alternatives and incentives. As with the previous published example of “best fit” framework synthesis [1], the *a priori* framework was found to accommodate most of the qualitative evidence from the included studies, enabling much of the extraction and synthesis to be completed rapidly and consistently by reviewers because the existence of the framework minimised the interpretive and iterative processes involved.

This initial coding was then supplemented by secondary thematic analysis of the remaining evidence that was not captured by the framework. This method applies the principles of standard thematic analysis [16] in the context of secondary research; it is distinct from thematic synthesis as there is no explicit line-by-line coding of the data [49]. The process involved the reduction of data into a small number of relevant themes which captured or reflected those data, and the exploration and description of those themes and their relationships. Individual reviewers independently interpreted the extracted data, assigned them possible themes, and then revisited those themes following familiarisation with the data and increasing numbers of studies. Themes originally assigned by an individual reviewer might therefore change or be refined during this iterative process, both to reflect the data more accurately and to capture similarities and differences within the data. The process is inductive, grounded in the data, and interpretive. Each reviewer therefore produced a list of new themes for data considered to be outside of the *a priori* framework. These themes and the data supporting them were then considered and discussed by the entire review team. A final consolidated list of new themes, and the data supporting or illustrating each theme, was then agreed by the review team after discussion of each reviewer’s own thematic interpretations (see Table 4). In this process the themes were “conceptualised” by being defined with reference to their supporting data, much like the development of the concepts in the *a priori* framework. The task of interpreting and conceptualising the “un-accommodated” data was not onerous as it only consisted of reaching consensus on the thematic categorisation and definition of a relatively small amount of data, with which each reviewer was already familiar and to which they had already assignedtentative new themes.

The reviewers produced six new themes for the case study framework, all of which related either to the roles and responsibilities of the employer in this area (employer obligations, employer responsibilities, and enforcement) or elements of the interventions themselves (ease and convenience; alternatives and cost; and co-worker support) (Table 4). The resulting new concepts, perhaps unsurprisingly, related to specifics of the setting (the workplace) and the interventions (components, delivery etc.), which were not well-represented in the more generic behavioural theories that formed the basis of the *a priori* framework. Many theories relate to personal health behaviours, but few theories relate to intervention components [50,51].

**Figure 5:**
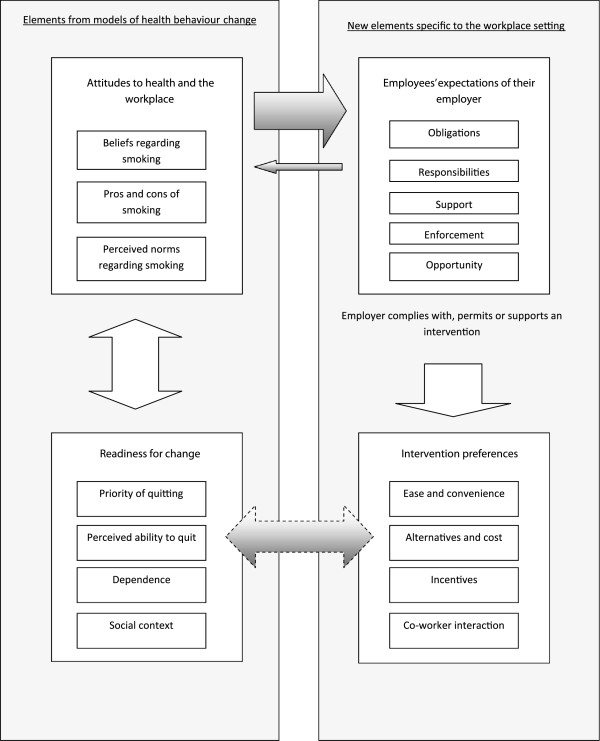
Conceptual model describing the nature of employees’ views of workplace smoking cessation or restriction interventions.

The revisions to the *a priori* concepts, and the creation of the new concepts, resulted from the reviewers’ interpretation of the data rather than the *a priori* framework, underlining the respective contributions of deductive and inductive analysis techniques. The resultant synthesis therefore built on the *a priori* framework, derived from all of the foundation models, but supplemented this framework with additional concepts reflecting the context, interventions and setting. The conceptual model resulting from the synthesis is depicted in Figure 5.

##### Stage 2: Creating the conceptual model or theory

The creation of the conceptual model or theory represents probably the most difficult process within the method to describe and runs the risk of reducing the creative nature of synthesis to a “mechanistic” task [52]. However, in principle the process relies on two stages of further reduction and interpretation of the data. First, concepts are clustered and subsumed as “internal attributes” within more abstract concepts [53]. In other words, where concepts shared commonalities, reviewer interpretation of the data reduced those characteristics to, in this case, four higher concepts relating to the behaviour of interest: *Attitudes to health and the workplace; Readiness for change; Employees’ expectations of their employer*; and *Intervention preferences*. So, for example, beliefs about smoking, pros and cons of smoking, and perceived norms regarding smoking were all clustered under the higher concept of *Attitudes to health and the workplace*. Second, these higher concepts and their “internal attributes” were each contextualised with reference to the data to understand their internal relationships [53]. For example, the attributes of “beliefs about smoking” and “perceived norms regarding smoking in the workplace” were strongly linked. Some smokers and non-smokers felt that there was no problem with smoking either at work or elsewhere [28,37]. Some non-smokers believed that smokers thought that smoking in the workplace was not an issue at all [25]. Some informants, smokers and non-smokers, held the opinion that smoking was no worse than many other hazards to which people were exposed at work and elsewhere [25,28]. In a similar fashion, perceived norms ranged from employees’ beliefs about their “rights”: The right to smoke in the face of bans or restrictions versus the right not to be exposed to others’ smoke in the workplace [25,29]. As a result of such different beliefs and norms, smoking and non-smoking groups would be formed within a workplace, with opposing perspectives, identities and aspects of community [25,28,29,38].

This two-stage process moved the analysis beyond lower–level interpretation or description of the data to a higher level of abstraction and theory creation, i.e. synthesis. The synthesis was then “expressed” through a simple diagram, as a conceptual model, reflecting the factors and relationships at work in the decision-making and behaviour of interest (e.g. Figure 5). This diagram was supplemented by a narrative referring to the actual studies and their data, to illustrate the complexities of the concepts and interactions within the model [15]. This enhanced the validity of the model by illustrating how the lower-level concepts or attributes had been derived from the data.

For example, the large left-to-right arrow between the concepts of *Attitudes to health and the workplace* and *Employees’ expectations of their employer* (see Figure 5) is explained by employees’ views about smoking and their employers’ responsibilities in complying with or supporting workplace smoking restrictions or interventions: “As far as opinions on the presence of smoking in the workplace are concerned, most workers believe (94.3%) that the employer should do everything to protect the non-smokers from having to inhale tobacco smoke” [37] or, the alternative view: "a few stated that their smoking was none of their employer’s business" [38]. However, it was also the case that some actions taken by employers, such as restrictions or bans, affected personal views about smoking and the workplace; this is represented by the smaller right-to-left arrow. One study reported the words of one smoker that, “I was a bit angry about it at the time, and I do think it was very much forced upon us. But having got used to it now, it’s actually not as bad as I thought it would be . . . I don’t feel as strongly now about it as I did then, because I can see the benefits” [28]; while in another study, participants commented about "management making it convenient to give up smoking" [30].

In the same way, the priority an individual smoker assigned to quitting was strongly related to views about specifics of the interventions. This is represented by the bi-directional arrow between the concepts of *Readiness for change* and *Intervention preferences*. If employers provided incentives and opportunity, this acted as additional motivation to those who wanted to stop smoking: “It was win-win. I wanted to quit anyways so you had the benefit of not smoking and getting paid not to smoke”, and, “It was the icing on the cake. It was a nice perk. I had been thinking about it (quitting) for a long time and it gave me a slight push” [36]. In other words, incentives and opportunity impacted on perceived ability to quit or likelihood of quitting. However, for those smokers without any such priority, incentives and opportunity made little or no difference: “I mean if you told me that I was going to make a million bucks if I quit in a year I guess I would be motivated to quit. But a few hundred bucks is not really a motivation” [36]. In other words, the priority an individual gave to quitting impacted on the likely effectiveness of any intervention and its components.

Thus, through the accompanying narrative the richness or “thickness” [54] of the data not only made it possible to demonstrate the origin of the various concepts and attributes, but also made it possible to articulate the relationships between them, and thus to create a new conceptual model. The resultant conceptual model can be used to generate a hypothesis or programme theory to develop interventions and be tested in empirical research. In this case, a working hypothesis might be that the priority given by an employee to quitting smoking mediates the effectiveness of any relevant workplace intervention [15]. Thus, the method, which begins by testing relevant theory within a specific context, can generate a refined, context-specific, intervention-based, programme theory that can itself by tested in empirical research.

#### Testing the synthesis: comparison with the *a priori* models, dissonance and sensitivity

The conceptual framework or model resulting from the synthesis must now be explored to assess the potential for bias (e.g. the unexplained absence of themes of known relevance, such as from the *a priori* framework, or if there are no negative cases within the evidence) and to determine if the synthesis is sensitive to variables such as the ajudged reported quality, design or location of included studies. When the synthesis is complete, any differences between the *a priori* framework and the new framework need to be explored. This would consist of explanations both for the absence of any *a priori* themes from the new framework and the presence of new themes. This needs to be completed in order to understand and contextualise the findings in relation to the foundation theories or models. It is also a test of publication bias within the sample of included primary research studies, i.e. is the absence of some *a priori* themes a reflection of understandable and reasonable differences between the *a priori* and final model on account of differences of setting, population etc. or does their absence need to be explored further by revisiting the literature. In this worked example all *a priori* themes found resonance in the evidence from the included studies. However, differences did exist in our previous published example of “best fit” and were explained by differences in the populations covered in the *a priori* and review frameworks [1]. In the smoking cessation example, the new themes added to the framework could be explained by the foundation models’ limited consideration of the variables of the intervention and setting, as noted above.

On a note related to publication bias, it has been argued that all qualitative evidence synthesis should involve a process of seeking the “disconfirming” [55] or “negative case” [54], as a means of testing the robustness, representativeness and validity of the evidence. We support this view that the presence of “uncomfortable” evidence should always be assessed in qualitative evidence synthesis. Indeed where such evidence is missing, purposive efforts should be made to identify possible disconfirming cases [55]. In this case study formal procedures to seek possible disconfirming cases were deemed unnecessary because multiple cases of dissonance, i.e. the presentation of contradictory views, were readily identified. For example, co-workers and family could act as a positive source of support and shared experience for employees trying to quit smoking, but the continued smoking of co-workers and family could also act negatively, as a barrier to someone being able to stop themselves [13]. The frequent presence of such dissonance, both within individual studies and the evidence as a whole, reflected the quality of the included studies and the depth of the evidence.

The authors also advocate that a qualitative sensitivity analysis be performed, following the synthesis stage, to examine the effect of variables, such as the quality of reporting of a study, within the qualitative evidence synthesis, i.e. how each individual study contributes to the final synthesis in terms of both “frequency” (the framework) and “thickness” (the model) [48]. The value and utility of some form of sensitivity analysis for qualitative reviews has been acknowledged elsewhere, i.e. in testing whether a synthesis is affected by the omission of studies with methodological flaws [56] or from certain sources [20]. In the case study reported here, 12 of the 15 studies clearly satisfied two or more of the four possible “quality” criteria illustrating the methodological processes conducted within the studies. These studies were therefore categorised as “Adequately reported”. Only three studies were categorised as “Inadequately reported” [30,34,37]. The two reviewers (JL, JR) applied these brief quality assessment criteria consistently, each independently categorising every study in the same way, i.e. as either adequately or inadequately-reported. In sensitivity analysis, following principles outlined elsewhere [48], the contribution to the synthesis of the three “Inadequately reported” studies was found to be limited. Exclusion of these three studies would not have affected the presence of any of the themes in the framework, or their depth (“thickness” [57]), complexity and relationships (as represented in the conceptual model). Only one inadequately-reported study [34] contributed anything unique: The view of some participants that the usability of self-help materials might help smokers to engage and be successful with an intervention, an idea not reported elsewhere within included studies. It is therefore likely that the exclusion of these potentially “lower quality” studies would not have adversely affected either the synthesis or the “thickness” of its detail. This echoes the findings of previous systematic reviews of people’s views that have undertaken such sensitivity analyses [49,58-60]. The same form of qualitative sensitivity analysis could be applied to explore whether a review’s findings were sensitive to other variables also, such as population, setting or location, study design or intervention. A sensitivity analysis based on quality, study design, setting and location was performed by the first author in a previous review and, again, the synthesis was found not to be affected by any of these variables [58].

These process elements of the “best fit” method, i.e. testing of the synthesis with reference to any “gap” between the findings and the original model(s), as well as an exploration of dissonance and a qualitative sensitivity analysis, were all conducted after completion of the synthesis and were undertaken with the express purpose of addressing concerns expressed elsewhere [61] that qualitative approaches to synthesis do not typically assess either the contradictions or the shortcomings of individual studies (in terms of their quality or “thickness”).

### Discussion

“Best fit” framework synthesis, as described and defined here, involves a series of distinct stages, each involving the application of a particular strategy or methodol. After the scoping of the question, common to all reviews, there follows two separate but simultaneous search and study selection processes. The BeHEMoTh strategy, described elsewhere (Booth A, Carroll C: Towards a simple transparent method for identifying theory for use in systematic reviews of behaviour change interventions: the BeHEMoTh Procedure. Submitted.), was used to systematically identify relevant models or theories relating to the behaviour and context of interest. Secondary thematic analysis of the model or models was then undertaken as a means of creating the *a priori* framework as the basis for the extraction and synthesis. In the previous worked example, this was based on a single published conceptual model, but in this present case study review there were five relevant models, which required reduction in order to produce the *a priori* framework. This process required more time to complete than for the single model, but was also worthwhile because the selection of only one of the adapted theories or models in the publications would have produced a more limited *a priori* framework. This can be deduced from the gaps in the matrix outlined in Figures 4. Indeed, this approach also enables an improved understanding of the ways in which existing theories might be lacking when it comes to answering certain review questions. Once the thematic framework was produced, the named themes were defined and conceptualised. A second search using the SPIDER strategy [21] was conducted to identify the primary research studies for the review. Like BeHEMoTH, this strategy was found to be fit for purpose for this exemplar review with the approach identifying 14 of the 15 included studies in the review.

**Figure 6:**
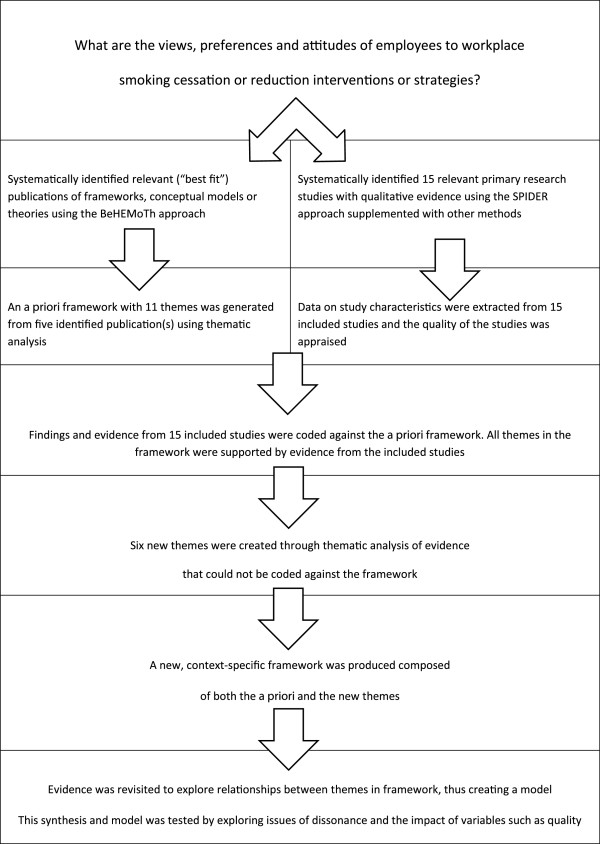
Worked example.

Reviewers conducted data extraction rapidly and consistently using the *a priori* framework with its defined themes. The review team then used secondary thematic analysis to interpret and analyse that evidence not captured by the *a priori* conceptual framework. Individual reviewers independently conducted the initial analysis and interpretation of this evidence, but these interpretations and evidence were then reassessed by the review team and a final list of themes agreed. This led to the creation of a new, agreed conceptual framework. Relationships between individual concepts were then explored with reference to the evidence, which, in turn, led to clustering of concepts and the creation of a new conceptual model describing and reflecting the behaviour of interest. Within the specific context of this review the result was a conceptual model explaining the behaviour and views of employees in response to workplace smoking cessation interventions. The resultant synthetic product was a model and, with further consideration and interpretation, a programme theory, whichcould be used to develop potentially more acceptable and effective workplace interventions for smoking cessation. The process, outlined in Figure 1, is described with reference to this particular review in Figure 6.

The quality assessment process, using the brief quality of reporting assessment criteria developed by the authors and used elsewhere [49], was also found to be fit for purpose for this review: The reviewers independently and consistently categorised each included study in the same way, as either adequately or inadequately reported. The final process elements of the methodology; the “gap analysis” with reference to the foundation models; the qualitative sensitivity analysis based on the relative quality of reporting of included studies; and the assessment of dissonance, all offer simple but important means of testing and evaluating the internal and external validity of the review and synthesis.

The “best fit” method does not apply any “saturation” criteria [62] and thus involves examination of evidence from all identified studies. It has been acknowledged that the threshold for saturation can be difficult to determine systematically [9,55] and may potentially ignore the contribution of later studies. This can prove a particular issue when conducting updates to pre-existing systematic reviews, especially where more recent studies have been found to offer greater depth and contribute more to a synthesis [48,63]. The two worked examples of “best fit framework synthesis” also suggest that it is not necessarily true in all cases to say that qualitative research linked to a specific intervention is of only “limited value” in generating conceptual models [62]: All of the included studies represented such research. The “best fit” method can therefore be useful, as it can test and generate theory, and in so doing solve problems in policy-making and health care, and answer those questions left unanswered by the quantitative paradigm. These are roles that are increasingly being recognised for qualitative health research [64-66] and, by extension, qualitative evidence synthesis.

The “best fit” method for systematic review and evidence synthesis, with its inductive approach to the testing, revision and supplementation of a foundation theory, satisfies some stated objectives of qualitative synthesis: To develop a precise view of theoretical knowledge within an area, as well as the gaps in that knowledge, and to provide an evidence-base for the future development of interventions [56]. This is because the method not only tests theory but can generate it. In this case study, it led to the creation of a “testable” hypothesis that the priority given by an individual to quitting smoking mediates the effectiveness of any relevant intervention [15].

#### Strengths and limitations of the “best-fit” method

“Best fit” framework synthesis is different from other approaches in that it is a theory-based qualitative evidence synthesis (akin to realist synthesis) but can employ more than one systematically identified model or theory for conducting framework synthesis, which might thus overcome the limitations of any one particular theory. It also employs a unique combination of framework synthesis and secondary thematic analysis. It is highly suited to generating context-specific models or theories of patient or client-behaviour or experience by utilising existing published models and theories..This is because of the existence of a large number of well-established theories and models explaining decision-making with regard to health and health care [67]. Consequently, even where there is not a “perfect fit” foundation model for a particular population and health behaviour, there will invariably be a generic model to act as a “best fit” and form the basis of the *a priori* framework. Such a framework will almost certainly accommodate a substantial amount of the evidence included in a review, given the universal traits of human behaviour and decision-making that influence health behaviours and are reflected in such models. As demonstrated here, specific workplace smoking cessation models were found and used, but all were based on more generic health behaviour models, such as the Health Belief Model (HBM) and the theory of planned behaviour (TPB). Generic models are likely to apply to most behaviours and are therefore likely to accommodate a large amount of the evidence from included qualitative studies, regardless of setting, population or intervention. The synthesis product should therefore be a conceptual model of value not only to researchers developing interventions, but also to policy and decision-makers. This is because it will not only be context specific and evidence-based, but also because it will draw heavily, and transparently, on existing theoretical foundations and traditions.

Another potential strength of the method lies in the inherent advantages of coding data by using both an *a priori* framework and the creation of new themes by applying inductive methods, where data cannot be accommodated by the framework. The approach therefore uses a framework but is not limited to it. Even though the specifics of the setting, population or intervention might fall outside of the scope of foundation models or theories, and therefore outside the *a priori* framework also, these aspects will be captured by the supplementary thematic analysis. This satisfies requirements of qualitative synthesis: “The researcher should … sort the data accordingly … be an active astute observer … so that … [they are] not forcing data … into prescribed categories … [but] rather … asking informed questions and checking the fit of established theory to see if it holds in a new situation” [68]. This dual role of qualitative researchers - to engage with theory but not be constrained by it – is acknowledged within the “best fit” methodology.

The value and utility of “best fit” for health services’ problem-solving and policy-oriented questions has already been recognised because it offers a theory-based synthesis method focusing on health behaviour and health service use [3,6], similar to realist synthesis [8]. However, this could be developed further were the technique to be used as a first stage in matrix-based synthesis methods to integrate qualitative and quantitative evidence to develop effective interventions [11,69,70]. This is especially the case with complex interventions and those where patient compliance (and thus the preferences and views of patients regarding services or technologies) are likely to be a mediator of optimal outcomes [11]. Health Technology Assessment is an obvious sphere for the application of “best-fit” [71]. It is even possible to generate context-specific, programme theory from this type of synthesis, which could then be tested in an empirical study. Further strengths, as identified previously, are the speed and consistency with which the process can be conducted and, unlike other more interpretive methods, such as meta-ethnography [9], the pragmatic requirement for a systematic review team to be technically competent but not necessarily to contain multi-disciplinary expertise. Consequently, the interpretive process of “best fit” framework synthesis, while more “mechanistic” than other forms of synthesis, is correspondingly more pragmatic and transparent.

However, the method is only appropriate for questions for which pre-existing frameworks or theories exist; the generation of completely new theory requires exclusively inductive methods, such as meta-ethnography or critical interpretive synthesis [5]. There is also the issue of the selection of relevant theory. A review team must make a decision on the relevance of any conceptual or theoretical papers identified based on the inclusion criteria and “best fit”. This case study identified five theoretical or conceptual papers, all considered to be equally relevant to the question and synthesis. Indeed, no one paper could have generated all of the themes of the *a priori* framework, thus demanding thematic analysis of a larger amount of evidence. Another review might identify even more potentially relevant model or theory papers. Unless a rationale was applied for selecting a small number of such papers, then the process of generating the framework could prove burdensome, when the intention behind the methodology and approach is actually to facilitate and simplify this stage. The best fit method makes use of a range of novel, published process elements for the systematic review and synthesis of qualitative evidence, namely: Systematic search strategies for models and theories, as well as for primary qualitative research studies; the assessment of study quality using simple, brief criteria; the application of framework synthesis methods to code much of the extracted data; the application of secondary thematic analysis to create both the *a priori* framework and the new themes in the final framework; and the post-synthesis application of forms of sensitivity and dissonance analysis. The innovative nature of so many elements of the methodology will obviously require extensive testing and evaluation. This paper represents a second attempt to specify and to develop further each stage of the “best fit” framework synthesis process within a worked example. Although all of the techniques applied appear to be fit for purpose, the method needs further testing by different groups and for different questions and health behaviours. However, this paper also provides a second, more developed “worked example” of the “best fit” method, and satisfies the call, made in this journal, for the publication of more worked examples of novel methods of evidence synthesis [11]. As noted elsewhere, such case studies provide a specificity and transparency often absent from original descriptions of methods [9].

### Conclusion

The “best fit” method of framework synthesis offers a pragmatic means of conducting rapid qualitative evidence synthesis and generating models and, potentially, programme theories, and is of potential relevance both to researchers and policy-makers. The method is suited to producing new conceptual models for describing or explaining the decision-making and health behaviours of patients and other groups and if effectiveness evidence is at all equivocal, then this method offers a means of exploring and explaining that ambiguity, and developing more appropriate interventions. The “best fit” approach applies new methods to identify theories in a systematic manner, and to create the *a priori* framework for the synthesis. Otherwise it uses an innovative combination of existing methods of quality assessment, analysis and synthesis to complete the process. The whole process was developed and tested within the context of a qualitative evidence synthesis of employees’ views of workplace smoking cessation interventions and was found to be both practical and fit for purpose.

### Competing interests

The authors declare that they have no competing interests.

### Authors’ contribution

CC and AB conceived and developed the "best fit" method; CC conceived and designed the case study; CC, JL and JR extracted the data, appraised included studies and analysed and interpreted the data. CC drafted the paper, AB, JL and JR undertook critical revision of important content of the manuscript. All authors approved the final version of the manuscript.

## Pre-publication history

The pre-publication history for this paper can be accessed here:

http://www.biomedcentral.com/1471-2288/13/37/prepub

## Supplementary Material

Additional file 1 Search strategies. Click here for file
