# Supplementary material for: “Best fit” framework synthesis: refining the method
Source: BMC Med Res Methodol. 2013 Mar 13;13:37. doi: 10.1186/1471-2288-13-37 (PMC3618126; doi:10.1186/1471-2288-13-37)
Supplement: Additional file 1 — Search strategies. [file 1471-2288-13-37-S1.docx]

**Additional file 1:**

**1: Example models search**

Database: PsycINFO <1987 to February Week 4 2012>

Search Strategy:

--------------------------------------------------------------------------------

1 exp Smoking Cessation/ (7383)

2 ((smoking or tobacco or health) adj2 (cess$ or quit$ or prevent$ or promot$)).tw. (22546)

3 workplace.tw. (16718)

4 (model$ or theor$ or framework or concept$).tw. (707340)

5 (regression or integrative model or integrative care model or economic or Markov or animal).tw. (155113)

6 1 or 2 (23786)

7 3 and 4 and 6 (207)

8 7 not 5 (181)

**2: Example primary research studies search**

Database: PsycINFO <1987 to October Week 4 2011>

Search Strategy:

--------------------------------------------------------------------------------

1 exp Smoking Cessation/ (7104)

2 ((smoking or tobacco) adj2 (cess$ or quit$ or prevent$ or promot$) adj6 ((work$ or employ$ or organ$) adj3 (intervention$ or incentive$ or promotion$ or program$ or support$))).tw. (106)

3 1 or 2 (7122)

4 (workplace adj4 ((health or lifestyle) adj3 promotion)).tw. (169)

5 exp Qualitative Research/ (2907)

6 (questionnaire$ or survey$ or interview$ or focus group$ or view$ or experienc$ or opinion$ or attitude$ or perce$ or prefer$ or qualitative).tw. (916483)

7 5 or 6 (916579)

8 3 and 4 and 7 (0)

9 workplace.tw. (16101)

10 1 and 9 (100)

11 2 or 4 (274)

12 10 or 11 (354)

13 7 and 12 (186)
